# Diagnostic Dilemma of Central Nervous System Tuberculosis with Neurocysticercosis and Neurosarcoidosis: A Case Report

**DOI:** 10.31729/jnma.8043

**Published:** 2023-02-28

**Authors:** Nikesh Bajracharya, Saral Lamichhane, Prakriti Lamichhane, Dirishya Bishowkarma, Ashirbad Acharya, Shanti Sharma, Prajwal Pandit

**Affiliations:** 1 KIST Medical College and Teaching Hospital, Mahalaxmi, Lalitpur, Nepal; 2 Shishuwa Hospital, Lekhnath, Pokhara, Nepal; 3 Sundarbazar Hospital, Sundarbazar, Lamjung, Nepal

**Keywords:** *brain*, *case reports*, *neurocysticercosis*, *sarcoidosis*, *tuberculoma*

## Abstract

Multiple ring-enhancing lesions are commonly encountered abnormalities in neuroimaging. There are many differentials for such lesions as infections, neoplasms, vascular lesions, inflammatory and demyelinating conditions, and granulomatous diseases. In developing countries, tuberculoma and neurocysticercosis are the two important etiologies to be considered. This case report illustrates how multiple ring-enhancing lesions can lead to our management in one direction while the true diagnosis remains elusive. A 53-year-old male who presented with a headache was initially diagnosed and treated as neurocysticercosis, then neurosarcoidosis ultimately turned out to be a case of Central Nervous System Tuberculosis on further evaluation. Consideration of only clinical scenarios and neurological imaging can lead to diagnostic inaccuracy, mismanagement and poor outcome, therefore, other supporting lab investigations should be considered for making a correct diagnosis.

## INTRODUCTION

Ring-enhancing lesions may indicate infections, neoplasms, and inflammatory or granulomatous diseases. Central nervous system (CNS) tuberculosis (TB) and neurocysticercosis are the two most common differentials to be considered in developing countries like ours.^1^ CNS TB presents with intracranial tuberculoma in 1% of CNS TB cases.^2^ Differentiating between intracranial tuberculoma and neurocysticercosis on the basis of clinical and radiological criteria have been proposed, but these are not absolute. Spontaneous resolution of ring-enhancing lesions is considered a major criterion for diagnosis of neurocysticercosis.^1^ Neurological involvement can be present in five percentage of sarcoidosis.^3^ One of the presentations of neurosarcoidosis in imaging is enhancing and nonenhancing parenchymal lesions in the brain.^3^

## CASE REPORT

A 53-year-old male presented to the outpatient department (OPD) with a history of headaches for one month. Headache was generalized, insidious in onset, mild, dull aching with no relieving and aggravating factors, and no other associated symptoms. There was no history of fever, night sweats, vomiting, altered sensorium, abnormal body movements, weight loss or any focal neurological deficit. The patient's history of contact with tuberculosis was negative. There was no significant past, family or travel history. On examination, he was well-built, conscious, cooperative, and well oriented to time, place and person. His vitals, and systemic, neurological and fundoscopic examination were normal. Signs of meningeal irritation were absent. Routine blood investigations were within the normal limit (Table 1).

**Table 1:** Routine blood investigations.

Laboratory test	Patient level
Hemoglobin	16.5 gm/dl
Total leukocyte count	9410 /cumm
**Differential count**	
Neutrophils	65%
Lymphocytes	22%
Eosinophils	3%
Monocyte	2%
Basophils	0%
Platelets	1,55,000 /cumm
Urea	20 mg/dl
Creatinine	0.8 mg/dl
Na+	130 mmol/l
K+	3.8 mmol/l
Blood sugar random	97 mg/dl
C-reactive protein	4 mg/l
Erythrocyte sedimentation ratio	8 mm/1^st^ hour

**Figure 1:**
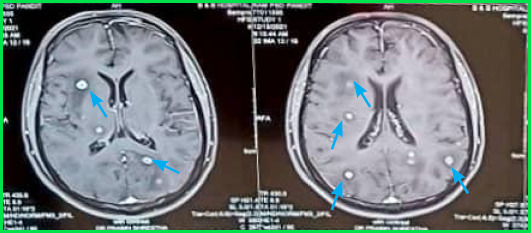
MRI brain (T1, coronal view) showing multiple ring-enhancing lesions.

**Figure 2:**
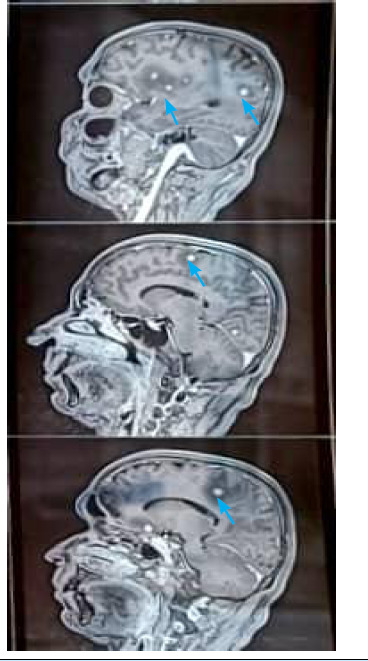
MRI brain (T1, sagittal view) showing multiple ring-enhancing lesions.

Chest X-ray appeared normal. Magnetic Resonance Imaging (MRI) of the brain showed multifocal monomorphic sub-centimetre-sized ring lesions with peri-lesional oedema diffusely distributed in the cortex and white matter of all lobes. All of these lesions showed avid ring enhancement post-gadolinium (Figure 1 and Figure 2).

Considering the clinical and laboratory findings, neuroimaging findings and geographical location of the patient, a probable diagnosis of neurocysticercosis was made and the patient was started on praziquantel, albendazole, anti-epileptics, and corticosteroid for two weeks.

However, on follow-up after two weeks, the patient's symptoms had not subsided so an MRI brain was repeated which showed lesions similar to that in the previous MRI. So, further workup was done to rule out other possible etiologies. Computed Tomography (CT) of the chest and abdomen was done that showed multiple miliary nodules in both lungs, tiny calcifications in the posterior segment of the upper lobe of the right lung and lateral basal segment of the lower lobe of the left lung, mild fibrotic changes in the inferior lingual segment and mildly enlarged paratracheal lymph nodes. CT chest and abdomen were suggestive of either tuberculosis or sarcoidosis. Sputum for Acid Fast Bacilli (AFB), sputum for gene Xpert, and Mantoux test were done for TB; and serum Angiotensin Converting Enzyme (ACE) was sent for sarcoidosis. Tests for TB were negative, however, the ACE level was raised, and hence a probable diagnosis of sarcoidosis was made. He was kept under prednisolone at a dose of 1 mg/kg/day. On follow-up after one month, he was symptomatically better so azathioprine was added as a steroid-sparing agent.

After three days of prednisolone plus azathioprine therapy, the patient had a fever which was sudden in onset, associated with chills and rigours, the maximum temperature recorded was 102.1°F. There was also a history of shortness of breath, acute in onset, progressive, continuous, and present even during rest. On examination, he was ill-looking, distressed and drowsy. There was an increased respiratory rate and oxygen saturation was found to be 46% in room air. On chest auscultation, breath sound was decreased bilaterally and crepitation was present in all the lung fields. Routine blood investigations and chest X-rays were done. Chest X-ray showed miliary mottling. The patient was admitted to the intensive care unit (ICU) where a bronchoscopy with biopsy was performed. A biopsy of the left lung lesion showed confluent fibro-histiocytic granulomas in the inter-alveolar spaces and alveolar wall. Those histiocytes were accompanied by mixed inflammatory cells. Ziehl-Neelsen (ZN) stain for AFB was positive. The patient was pathologically confirmed to be a case of TB and was started on anti-tubercular therapy, following which the patient gradually improved. The patient has completed four months of anti-tubercular therapy and the patient is getting symptomatically better.

## DISCUSSION

Multiple ring-enhancing lesions in the brain have myriad differential diagnoses and getting a definitive diagnosis out of these is a challenge. In a prospective study conducted in India, infections (39%) and neoplasms (16%) were the most common causes of multiple ring-enhancing lesions in the brain. TB followed by neurocysticercosis were the commonest infective pathologies responsible for such lesions.^1^ However, in Western countries, non-infective causes (brain malignancies: gliomas (40%) and metastasis (30%)) were the most common causes for ring-enhancing lesions in the brain as per one of the studies.^4^ Due to close geographical proximity with India, and similar lifestyles, even in our country Nepal, TB and neurocysticercosis are very common.^5^ So, TB and neurocysticercosis must be highly considered as differentials for multiple ring-enhancing lesions in our settings.

However, due to the lack of specific symptoms directing to one or the other, it is challenging to make a definite diagnosis clinically between TB and neurocysticercosis.^6^ Both TB and neurocysticercosis may present with nonspecific symptoms like headache, seizures, and signs of raised intracranial pressure like altered sensorium and vomiting.^2,7,8^ Therefore, clinically it is very hard to differentiate between these two conditions. On top of it, sarcoidosis may present as ring-enhancing lesions in the brain on rare occasions, which may create more diagnostic confusion.^3,9^

For neurocysticercosis, serum cysticercus IgG antibody (by enzyme immunoassay) is used. Recently monoclonal antibody test ELISA are used to detect Taenia solium antigens in urine which has a sensitivity of 92% for viable parasites.^10^ Studies have indicated the use of magnetic resonance spectroscopy (MRS) to guide towards the diagnosis.^11^ However, in low-resource countries like ours, such facilities are not easily available and the diagnosis moreover depends on the clinical judgement, which may be wrong in many cases. In this case, our patient who presented with headache was initially diagnosed as a probable case of neurocysticercosis according to 'revised diagnostic criteria for neurocysticercosis'.^11^ Patients also got treated as a case of neurosarcoidosis due to diagnostic inaccuracy. Had there been the availability and accessibility of proper diagnostic tools, the correct diagnosis could have been made which would have led to early and proper management of the patient.

Diagnostic inaccuracy leads to mismanagement and poor outcome as the treatment of neurosarcoidosis and neurocysticercosis are completely different from one another, and at times wrong treatment can flare up the primary disease. Hence, close follow-up after empirical treatment of one is imperative such that errors if they occur can be corrected at the earliest.

This case highlights the diagnostic dilemma created by the multiple ring enhancing lesions in the brain, and also warns about the consequences of mismanagement that occur due to diagnostic inaccuracy. Besides TB and neurocysticercosis, sarcoidosis can also confuse the treating physician with multiple ring-enhancing lesions. A comprehensive clinical picture, the immunological status of the patient, geographical location, and radiographic imaging are important factors to be considered for making a correct diagnosis. Looking after other organ systems may also lead us towards some diagnostic clues which might have not been sought with our focus solely on the neurologic symptoms.

## References

[1] Garg RK, Desai P, Kar M, Kar AM (2008). Multiple ring enhancing brain lesions on computed tomography: An Indian perspective.. J Neurol Sci..

[2] Mohammadian M, Butt S (2019). Symptomatic central nervous system tuberculoma, a case report in the United States and literature review.. IDCases..

[3] Naqi R, Azeemuddin M (2012). Case Report Neurosarcoidosis.. J Pak Med Assoc..

[4] Schwartz KM, Erickson BJ, Lucchinetti C (2006). Pattern of T2 hypointensity associated with ring-enhancing brain lesions can help to differentiate pathology.. Neuroradiology..

[5] DoHS. (2020). Annual Report DoHS 2019/2020..

[6] Rajshekhar V (2016). Neurocysticercosis: Diagnostic problems & current therapeutic strategies.. Indian J Med Res..

[7] Verma R, Gupta R (2014). Multiple ring-enhancing lesions: diagnostic dilemma between neurocysticercosis and tuberculoma.. Case Reports..

[8] Talukdar B, Saxena A, Popli VK, Choudhury V (2002). Neurocysticercosis in children: Clinical characteristics and outcome.. Ann Trop Paediatr..

[9] Bradshaw MJ, Pawate S, Koth LL, Cho TA, Gelfand JM (2021). Neurosarcoidosis pathophysiology, diagnosis, and reatment.. Neurol Neuroimmunol Neuroinflammation..

[10] Del Brutto OH, Nash TE, White AC, Rajshekhar V, Wilkins PP, Singh G (2017). Revised diagnostic criteria for neurocysticercosis.. J Neurol Sci..

[11] Pretell EJ, Martinot C, Garcia HH, Alvarado M, Bustos JA, Martinot C (2005). Differential diagnosis between cerebral tuberculosis and neurocysticercosis by magnetic resonance spectroscopy.. J Comput Assist Tomogr..

